# Regional Cultivation Forms of *Hericium erinaceus* Across China’s Climatic Zones: A Scoping Review and Analytical Lens for Climate-Adaptive Production

**DOI:** 10.3390/jof12040285

**Published:** 2026-04-17

**Authors:** Dongting Yang, Lin Zhu, Qiaoping Zheng

**Affiliations:** Lishui Institute of Agriculture and Forestry Sciences, Lishui 323000, China; dtyang@zju.edu.cn (D.Y.);

**Keywords:** *Hericium erinaceus*, climate-resilient agriculture, agroforestry, germplasm–cultivation–processing–market (GCPM) integration, nutraceuticals, regional adaptation

## Abstract

The cultivation of *Hericium erinaceus* in China accounts for about 85% of the global supply. Its decentralized production systems have developed across diverse climate zones, leading to distinct, location-specific practices recorded in local technical standards. This scoping review synthesizes these empirical protocols from five agro-climatic regions. It illustrates how adaptive strategies such as cold-tolerant strains in the northeast and market-driven precision in the subtropics are associated with yield stability. These practices reflect two interconnected forms of diversity. One is the diversity of cultivation systems themselves, from forest-based methods to industrial-scale production systems. The other is the diversity of locally adapted strains developed for specific environments. We use the Intelligent Germplasm–Cultivation–Processing–Market (GCPM) Integration framework to connect local practices with broader questions of systemic resilience. The evidence draws on field-validated standards, not controlled experiments, reflecting the current state of research. This work presents China’s practical knowledge as a reference for designing context-sensitive, climate-resilient cultivation systems elsewhere, suggesting that resilience may depend more on intelligent adaptation to local conditions than on one-size-fits-all solutions.

## 1. Introduction: Global Demand, Chinese Expertise, and the Knowledge Barrier

The global pivot towards preventive health and scientifically backed wellness products has positioned functional mushrooms as a prominent sector [1]. Furthermore, in the context of accelerating global climate change, developing climate-resilient cultivation systems for high-value crops has become an urgent agronomic and ecological imperative [2]. This aligns with the Food and Agriculture Organization’s Strategic Framework 2022–2031, which emphasizes the transformation to more efficient, inclusive, resilient and sustainable agrifood systems through the “Four Betters”: better production, better nutrition, a better environment, and a better life. Within this framework, climate-change-mitigating and adapted agrifood systems represent a key Programme Priority Area, supporting member countries to adopt innovative solutions for climate resilience [3]. Among such solutions, *Hericium erinaceus* distinguishes itself through a distinctive combination of culinary appeal and a robust, expanding body of pharmacological evidence [4]. Its bioactivity is primarily attributed to unique *H. erinaceus* polysaccharides (HEPs) and the rare Erinacines, Hericenones. These components have been linked to cognitive enhancement, neuroprotection, and gut–brain axis modulation [5,6,7]. This has elevated its status to a mainstream dietary supplement and therapeutic candidate. Recent advances in post-harvest processing further suggest this potential [8]. For instance, bioactive peptides can be extracted from *H. erinaceus*, such as KSPLY (Lys-Ser-Pro-Leu-Tyr). These peptides have shown immunomodulatory effects, which are achieved through mechanisms like TLR4/NF-κB pathway activation and macrophage polarization, and they have high bioavailability for functional food applications. This dual-purpose nature is widely regarded as contributing to its commercial value and has been associated with growing international demand for reliable, high-quality, and scalable production systems. This demand coincides with the ongoing transition of China’s edible fungus sector, which is shifting from traditional cultivation toward a fully integrated, intelligent, and value-added industrial chain [9]. The Intelligent Germplasm–Cultivation–Processing–Market (GCPM) Integration framework conceptualizes mushroom production as an interconnected continuum linking genetic resources, cultivation practices, post-harvest processing, and market dynamics, thereby supporting systemic resilience. China’s decentralized adaptive strategy integrates fungal cultivation with agroforestry, crop residues and forest ecosystems [10]. Compared with monocropping, such mixed farming systems are often associated with practices that may contribute to climate resilience, a characteristic that coincides with their coordination of advancements from germplasm innovation to market development.

**Figure 1:**
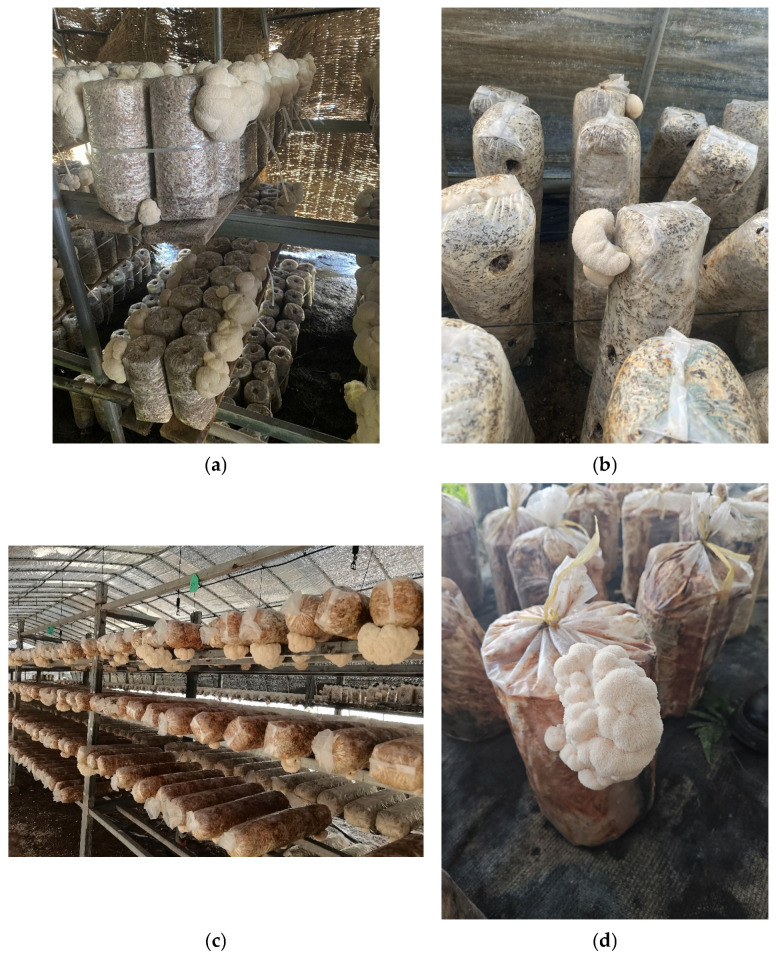
Representative cultivation forms of *H. erinaceus* in China. (**a**) Northeast China: Short bags (polypropylene, 170 × 370 mm, 0.055 mm thick) filled with substrate (cottonseed hulls, sawdust, bran) on steel racks; bag height 210–220 mm after filling. (**b**) Southern China: Long bags (LDPE, 130 × 550 mm, 0.005 mm thick) inclined at 15–30° on wire supports 20–30 cm above ground in shaded greenhouse. (**c**) Southern China: Long bags (LDPE, 130 × 550 mm, 0.005 mm thick) on multi-tiered racks with inoculation holes downward in shaded greenhouse. (**d**) Southern China: Wood segments (15–30 cm long, 1–5 per bag) in polyethylene bags (550 × 300 mm, 0.06–0.08 mm thick) placed directly on ground in shaded greenhouse; ends tied after sterilization.

The cultivation of *H. erinaceus* in China has a centuries-long history, preceding its modern scientific validation. Traditionally recognized for its distinctive morphology and medicinal value, it was documented in ancient materia medica for its benefits to digestive and nervous system health and was historically offered as tribute to imperial courts. This historical significance as a luxury and therapeutic resource now informs a substantial modern industry. This historical value has driven extensive informal observation over centuries. More recently, it has contributed to decades of systematic refinement. These efforts have contributed to the development of highly detailed, region-specific technical protocols. China plays a leading role in the global supply chain, accounting for an estimated 85% of annual output. This is attributed to a decentralized, highly adaptive agricultural strategy. This strategy translates ancient reverence into modern practice. Production is distributed across an exceptionally diverse climatic gradient, from the sub-arctic winters of Heilongjiang to the oxygen-thin heights of the Tibetan Plateau. This geographical distribution has been accompanied by highly distinct, location-specific cultivation methodologies, each encapsulated within local technical standards. These are illustrated in Figure 1 as four representative cultivation forms. These protocols represent deliberately redesigned cultivation architectures where every element is precisely specified in response to local environmental pressures and resource availability. The pre-harvest phase is a key critical leverage point for determining both yield and the qualitative attributes that define market value [11,12,13,14,15,16]. It encompasses the entire sequence from germplasm selection and substrate formulation, through spawn running and primordia initiation, to just before harvest.

Paradoxically, this wealth of practical, field-validated knowledge exists in a state of parallel isolation to the mainstream international scientific literature. There is a conspicuous imbalance in academic research. It focuses disproportionately on post-harvest extraction and bioactivity validation. In contrast, the foundational pre-harvest agronomy remains largely uncharted in Western journals. This gap is exacerbated by a significant ‘knowledge barrier’ that vital detailed technical specifications are documented in Chinese local technical standards and industry monographs, resources often linguistically and institutionally inaccessible to the global community. As a result, new regions often rely on generic, non-adapted cultivation methods.

This scoping review seeks to organize and present the localized cultivation knowledge of *H. erinaceus* from across China, offering a structured account of practices that have remained largely inaccessible to the international community. We employ a “climatic zone adaptation” perspective to classify the core pre-harvest strategies documented in various regions. Our aim is threefold. First, to compare these practical approaches. Second, to discuss related innovations. Third, to explore the role of the Intelligent Germplasm–Cultivation–Processing–Market (GCPM) conceptual framework [10]. This framework can serve as a conceptual lens to understand the cultivation-value chain as an interconnected system.

Popular and non-scientific knowledge, often documented in gray literature, plays a crucial role in driving scientific innovation by providing field-validated insights that may precede formal research [17]. Gray literature encompasses various forms of practical knowledge, including technical reports, government documents, and industry manuals that are not controlled by commercial publishers [18]. Including such literature in narrative and scoping reviews is essential for capturing a complete picture of a research field, particularly in areas where peer-reviewed studies are scarce [18,19].

At its heart, this work is an exercise in knowledge translation. We primarily regard the GCPM framework as a useful analytical lens. It serves to connect the dots between empirical practices in germplasm, cultivation, processing, and market. It is not fully validated model. A key premise of this review is that China’s extensive, protocol-driven experience represents a valuable repository of practical adaptation knowledge. We believe this knowledge deserves wider recognition and discussion.

China’s diverse climatic zones have not only shaped region-specific cultivation practices but also preserved and generated significant *Hericium* diversity, spanning cultivation systems, species, and phenotypic traits. From wild relatives in plateau ecosystems to locally adapted cultivars in temperate and subtropical zones, this diversity represents both a biological resource and a key element of climate-resilient production. By synthesizing region-specific practices and proposing the GCPM framework, we aim to highlight how fungal diversity, in its various forms, can be harnessed for sustainable biotechnology applications.

Therefore, this work seeks to offer two main contributions. First, it synthesizes this dispersed practical knowledge into a more accessible format. Second, it offers the GCPM framework as a reference point for further discussion. We hope this synthesis can serve as a useful reference and a starting point for dialogue. It is especially intended for cultivators, extension specialists, and researchers worldwide who are interested in the principles of climate-resilient production. We hope this work contributes to a broader conversation on how localized solutions can inform the sustainable development of cultivation systems globally.

## 2. Review Scope and Synthesis Methodology

This review is informed by the methodological framework for scoping reviews outlined in the JBI Manual for Evidence Synthesis [20] and follows the guidance of the PRISMA extension for Scoping Reviews (PRISMA-ScR) [21]. The scope was defined using the PCC (Population, Concept, Context) framework as follows:

Population: Cultivators of *Hericium erinaceus* in China, including local experts, technicians, and producers across different scales.

Concept: Cultivation techniques, region-specific substrate formulations, pest and disease management practices, phenotypic variability of strains, and the inclusion of other *Hericium* species.

Context: Five distinct climatic zones in China (cold temperate, temperate, subtropical, southwestern transitional, and plateau), with consideration of local climatic factors and resource availability.

**Search and selection of gray literature** 

We searched for Chinese local technical standards related to *Hericium erinaceus* in three databases: CNKI (*n* = 4), Wanfang (*n* = 55), and the National Public Service Platform for Standards Information (*n* = 36), yielding 95 initial records. After removing duplicates and excluding documents unrelated to cultivation (such as processing standards, product specifications, or superseded versions), 15 technical reports remained for full-text assessment.

These 15 reports were evaluated using the following inclusion criteria: (i) relevance to established production regions; (ii) documentation of practices with at least five years of commercial application; and (iii) sufficient agronomic detail for cross-regional comparison. Reports were excluded from Table 1 if they lacked region-specific detail, did not reflect distinct climatic characteristics, or had limited technical distinctiveness or low regional production volume.

All 15 reports informed the overall synthesis and knowledge gap identification. From these, eight representative standards were selected for detailed comparison in Table 1, chosen to illustrate the diversity of cultivation practices across the five climatic zones, with each selected report corresponding to a distinct production region and providing a full set of agronomic parameters. The remaining seven reports contributed to the narrative synthesis and regional practice descriptions throughout the manuscript.

**Critical mapping and evidence synthesis** 

Following JBI guidance, we summarized key cultivation variables by region. Table 1 presents a comparative synthesis of substrate formulas, sterilization parameters, environmental setpoints, and pest management strategies as documented in the original standards. The table note acknowledges the inherent heterogeneity of the source materials and positions this synthesis as a practice-based reference rather than a dataset for cross-zone statistical comparison.

The synthesis identifies knowledge gaps where cultivation practices have not yet been validated through experimental science. This aligns with the scoping review objective of mapping existing knowledge and identifying directions for future research [20].

**Figure 2:**
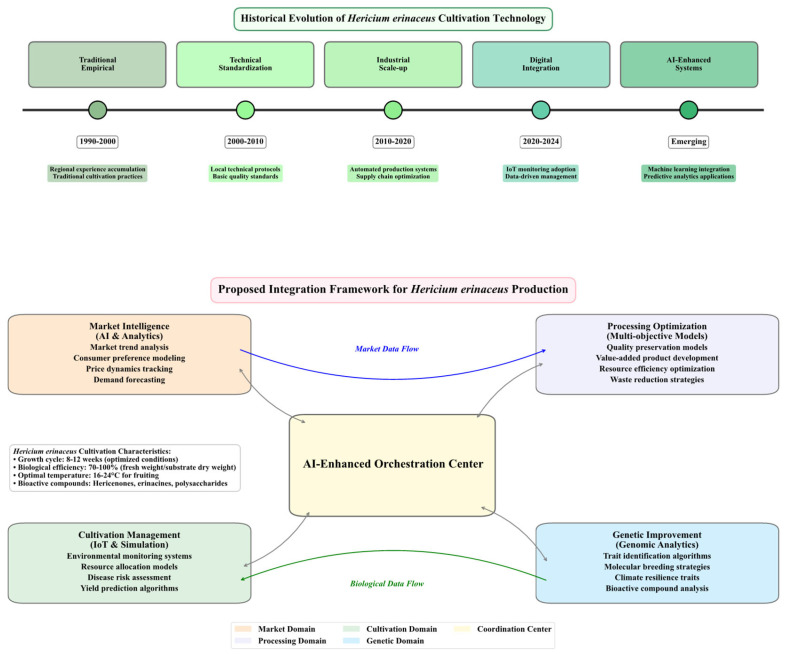
Historical evolution and the GCPM integration framework as an analytical lens for *H. erinaceus* cultivation systems. (**Upper**) Timeline of Chinese cultivation phases. (**Lower**) GCPM framework with AI-driven orchestration center optimizing market, cultivation, processing, and genetic domains under sustainability constraints.

The GCPM framework (Figure 2) is employed here as an analytical lens to organize this diverse information, rather than as a validated model. It is used to highlight interactions between germplasm selection, cultivation management, post-harvest considerations, and market linkages.

We note that this is a qualitative synthesis, bringing together typically siloed knowledge sources, including academic literature and extensive gray literature.

## 3. Climate-Zone-Specific Cultivation Systems: A Synthesis of Empirical Protocols

**Figure 3:**
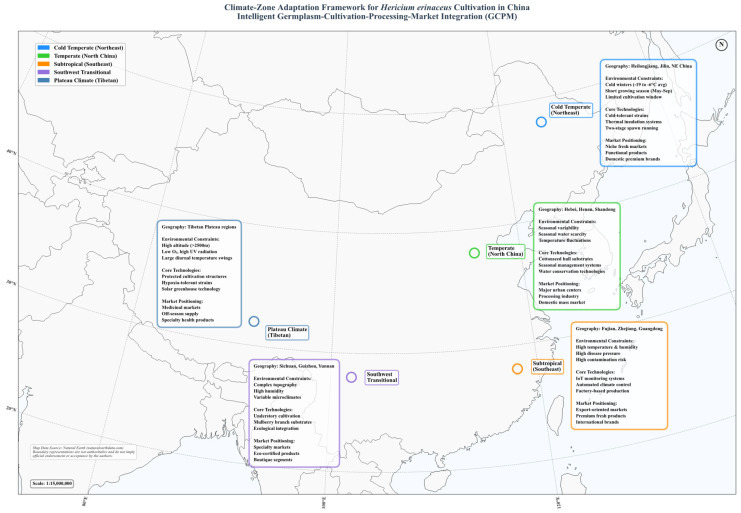
Climate-zone adaptation GCPM framework for *H. erinaceus* cultivation in China.

The cultivation form of *H. erinaceus* in China can be viewed as an example of biological management through environmental mediation. We suggest that its effectiveness stems not from applying a universal recipe, but rather from executing a systematic, climate-informed redesign of the entire pre-harvest continuum. The essence of these geographically distinct systems is distilled through an integrated lens covering germplasm, cultivation practice, and market linkage. These are conceptualized in our Climate-Zone Adaptation framework (Figure 3). We use this framework to explore the intricate interactions that shape production across China’s diverse ecologies, with specific parameters drawn from regional technical protocols. The detailed analysis of the five distinct production zones illustrates a consistent pattern of climate-informed cultivation design. To facilitate a direct comparison and provide a concise technical overview, Table 1 synthesizes the core agronomic parameters extracted from the key local technical reports governing each form. This comparative compilation illustrates how substrates, environmental controls, and management strategies are adapted to local climatic pressures and resource endowments. It thus moves beyond isolated description to form an operational framework.

Before examining region-specific practices, it is useful to outline the general cultivation process for *H. erinaceus*. The cultivation typically involves several key stages. First, substrate is prepared from locally available materials such as sawdust, cottonseed hulls, or agricultural residues, often supplemented with bran and minerals. The mixture is then sterilized, either through atmospheric steaming or high-pressure autoclaving, to eliminate competing organisms. After cooling, the substrate is inoculated with spawn (mycelium-colonized grain or sawdust) and allowed to colonize during the spawn running phase. Once colonization is complete, environmental conditions, including temperature, humidity, light, and CO_2_, are adjusted to induce primordia formation and subsequent fruiting body development. Finally, mature mushrooms are harvested, processed, and marketed. While this general sequence applies across regions, the specific parameters at each stage are finely tuned to local climatic conditions and resource availability.

### 3.1. Cold Temperate Zones: Thermal Efficiency Strategies

In the cold temperate realms, exemplified by the Hailin region of Heilongjiang, the overarching cultivation challenge is one of energy conservation and thermal stability against a backdrop of prolonged, severe winters. Here, winter temperatures may frequently drop below −30 °C, creating two distinct pressures: first, the need for strains that can survive mycelial dormancy at sub-zero temperatures; second, the requirement for energy-efficient infrastructure to maintain fruiting conditions without prohibitive heating costs. The foundational strategy, a core tenet of intelligent germplasm selection, is the deployment of fungal strains empirically selected for marked cold tolerance. Technical protocols from Hailin, such as the official report for the geographical indication product “Hailin Hou Tou Gu” [22], highlight cold-tolerant strain characteristics critical for local conditions. The local strains like ‘Heiwei 9910’, ‘Xing’an Houtou 1’, and ‘Mu Hou 1’ have been variety certificated and widely adopted through generational practice and annual technical guidance from local agricultural authorities. These locally validated strains can withstand temperatures as low as −2 °C to 0 °C during mycelial dormancy and initiate stable fruiting body development within the narrow, cool window of 14–18 °C. This biological adaptation is complemented by a distinctive regime of thermal management. It often uses a two-stage spawn running process to generate metabolic heat, along with specialized infrastructure such as semi-underground mushroom houses with double-layer insulated walls and integrated heating systems (e.g., underfloor heating maintaining water temperature at 40–45 °C, coupled with hanging radiators).

Substrate formulation in these zones is a direct reflection of local resource endowments, predominantly leveraging the abundant coniferous and broadleaf forests. While various broadleaf sawdusts are recommended by the local protocol, in practice, sawdust from oak (*Quercus mongolica*) and other *Fagaceae* species is often prioritized as the carbon backbone of the substrate (comprising 70–78% or more of the mixture) due to its particular suitability for cool-climate cultivation. This is balanced with nitrogen supplements such as wheat bran (15–20%), to achieve a C/N ratio of 25:1 to 30:1. This ratio is calibrated to support the slower metabolic rate at lower temperatures. The management of substrate moisture is particularly nuanced; Hailin’s technical standards mandate a lower moisture range of 58–62%, a key measure to mitigate the risks of mold contamination in less evaporative conditions. The entire physical infrastructure serves a single goal: the retention of metabolic heat and the stabilization of the microclimate. However, this system may illustrate its primary vulnerability, which is a marked dependency on a specific, localized resource base. The Hailin model, characterized by its large-scale, high-yield production, is closely tied to the broad-leaved-dominated forest ecosystems of Northeast Asia. This localized resource dependency mirrors a broader pattern in intensive agriculture, where high-yielding systems often become tightly coupled to specific, non-renewable or geographically concentrated inputs [23]. For instance, the modern “Green Revolution” cereal production paradigm achieved massive yield gains through a deep dependence on synthetic fertilizers (particularly phosphate and potash from limited global deposits), improved irrigation, and fossil fuels for mechanization and transport [24,25]. Similarly, the productivity of the Hailin system relies on a specific biotic resource: broadleaf forest biomass. Although renewable, this resource is spatially fixed and faces its own sustainability pressures. This highlights a recurring theme of resource dependency in regionally optimized systems where high productivity is achieved through intensive, localized inputs. It serves as a key consideration for any Cultivation–Market integration that depends on specific, non-universal resources. This case illustrates a broader principle in climate-resilient design: high optimization for specific local conditions often entails deep dependency on localized resources, a critical trade-off that could be managed in any regional adaptation strategy. This tension between specialization and resilience is not unique to agriculture but is a basic principle in ecology and evolutionary biology [26]. Specialist species evolve highly efficient traits to exploit a specific niche (e.g., the giant panda’s dependence on bamboo). They gain a competitive advantage within that niche, but become vulnerable to environmental changes that affect their key resource. Conversely, generalist species maintain broader resource use and habitat tolerance, often at the cost of peak efficiency in any one setting [27]. The Hailin model, akin to a cultivation “specialist,” has evolved high performance for its cold temperate, forest-rich niche. Understanding this trade-off through the lens of ecological specialization theory provides a perspective for assessing the long-term resilience of regionally optimized cultivation systems. These locally adapted strains and cultivation protocols are associated with fruiting bodies with distinctive nutritional profiles (e.g., higher polysaccharide and protein content) suitable for nutraceutical processing and functional food development.

### 3.2. Temperate Zones: The Dynamics of Seasonal Management

The central temperate zones, including provinces like Henan and Hebei, present a contrasting set of challenges defined by pronounced seasonal and diurnal fluctuations in temperature, coupled with characteristically low atmospheric humidity (may fall below 30% in spring). These conditions require strains with broad thermal plasticity rather than extreme cold tolerance, and management strategies that can buffer against rapid temperature shifts while maintaining surface humidity to prevent fruiting body desiccation. In these zones, cultivation strategies focus on managing variability and leveraging natural thermal cycles rather than mitigating a single consistent stressor. Local technical guidance [28] emphasizes broad thermal plasticity as a key adaptive trait for strains in this region. Varieties that can thrive across a wide temperature range (10 °C to 30 °C) have been empirically selected. These varieties maintain stable expression of key hydrolytic enzymes for efficient substrate decomposition, even under fluctuating temperatures. This biological preparedness contributes to the region’s distinctive cultivation rhythm.

The substrate strategy, detailed in regional protocols, reflects agro-industrial synergy and circular economy principles, capitalizing on the region’s status as a major crop producer. Abundant local agricultural by-products are leveraged in typical formulas [29] with cottonseed hulls (50–60%) and crushed corn cobs (20–30%) serving as the primary carbon sources. This integration represents a mixed farming system where fungal cultivation synergizes with local crop agriculture, potentially contributing to landscape resilience while significantly reducing both cost and the ecological footprint associated with dedicated forestry harvests. The C/N ratio is adjusted to a slightly higher 30:1 to 35:1 to modulate nutrient release kinetics, matching the variable metabolic demands across seasons. However, the efficiency of this “waste-to-food” model is contingent upon a stable, proximate, and qualitatively consistent supply of these specific agricultural residues. This tight coupling links the mushroom industry’s viability to local cotton and corn systems, including their market dynamics, pest management, and cropping patterns. This illustrates how cultivation systems are co-shaped by adjacent agricultural markets and vulnerable to them [30]. The substrate strategy in temperate zones, relying on cottonseed hulls and corn cobs, contrasts sharply with the sawdust-based formulas of the cold northeast. This shift from forestry by-products to agricultural residues reflects not only local resource availability but also a different form of climate adaptation: while the northeast depends on local forest biomass for thermal efficiency, temperate systems on the North China Plain leverage farmland crop residues to buffer against market volatility in the agricultural sector.

Cultivation management is inherently calendrical, responsive, and micro-climatically nuanced. Technical schedules prescribe spring inoculation (March-April) to align spawn running with rising temperatures, and autumn starts (September) to utilize residual warmth for colonization before the cool, high-quality fruiting period. Critically, the large diurnal temperature variation and low ambient humidity characteristic of the North China Plain are actively managed. Growers utilize these cool, dry nights to stimulate primordia formation and employ fine-droplet misting or micro-sprinkler systems within enclosed sheds to maintain the key 85–95% relative humidity at the fruiting body surface, preventing desiccation and cracking. This deep, place-based knowledge, while highly effective, is indicative of the empirically optimized yet input-sensitive nature of the current system [31,32]. This points to a notable opportunity for intelligent integration. Traditional scheduling can be augmented with predictive models that combine real-time weather, substrate telemetry, and strain-specific physiological data. This may further reduce production risks under increasing climate volatility [33].

### 3.3. Subtropical Zones: Industrial Precision, Market Proximity, and the Value Chain Imperative

The subtropical production hubs of southeastern China, epitomized by Gutian in Fujian and Changshan in Zhejiang, represent a key advanced form of industrial-scale, technology-intensive fungal cultivation. These systems are evolved not only to survive, but to productively use long growing seasons. The region faces dual bioclimatic stressors: sustained high temperatures (>30 °C for 3–4 months annually) and high relative humidity (>80% year-round). These warm, humid conditions create an ideal environment for competitive fungi and pests. This is consistent with this zone having invested most heavily in environmental control technologies. The biological foundation is the selection of explicitly thermotolerant and humidity-resilient strains, such as ‘Changshan 99’ and ‘Zhelin Hou 2’, bred and validated for vigorous mycelial growth and reliable pinning under these stressful conditions. Bag specifications vary across China’s production regions. In the cold northeast (Hailin) and on the high plateau (Tibet), thicker polypropylene bags (0.05–0.055 mm) are commonly used. These bags are suitable for high-pressure sterilization, which is often adopted in larger-scale operations. In subtropical and temperate zones, thinner bags (0.004–0.005 cm, approximately 0.04–0.05 mm) are more common. These may be made of polyethylene for atmospheric sterilization or polypropylene for high-pressure sterilization, depending on local practices. The long bags (55 cm) frequently used in southern China have very thin walls. This may help with heat dissipation during the warm growing seasons. Overall, the variation in bag thickness from north to south is consistent with what might be expected as a response to local thermal conditions. However, other factors such as sterilization methods and production scale could also influence these choices. As illustrated in Figure 1, this contrast between thick polypropylene in the cold northeast and ultrathin LDPE in the hot, humid south suggests a tangible link between cultivation practices and local thermal conditions.

The relentless pressure to mitigate biological risk and ensure crop security has motivated an unprecedented level of environmental control, now often integrated with renewable energy solutions. Real-time, sensor-driven management is mandated by protocols [34,35]: ventilation is modulated by variable-frequency fans in response to CO_2_ levels, and water curtain cooling is activated at precise temperature thresholds. An emerging and symbolic innovation in this region is the ‘photovoltaic mushroom greenhouse’ [36,37]. Here, rooftop solar panels serve a dual function: generating electricity for environmental controls and providing shade. They reduce indoor heat load by 3–5 °C and directly lower cooling energy demand. This represents a tangible step toward mitigating the system’s “sustainability paradox.”

Critically, this shift toward capital-intensive precision is strongly shaped by favorable market geography. The region is close to the affluent, dense urban clusters of the Yangtze and Pearl River Deltas, within a 48 h fresh supply chain. This shifts the core goal from bulk yield to guaranteeing consistent high quality, safety, and visual appeal demanded by premium retail and export channels. Consequently, cultivation parameters are strongly influenced by downstream market preferences: substrates shift towards ≥90% certified organic ingredients to meet traceability standards, and light spectra are fine-tuned to preserve the pale coloration prized by consumers. The emphasis on organic substrates and precise light control may not only meet premium market demands but also ensure consistent food safety and sensory quality for fresh and processed products.

However, the southeastern model may suggest a pivotal dichotomy between technological capability and value chain equity. While its automation is advanced, field observations present a suggestive counterpoint: in major cold temperate production bases like Hailin, Heilongjiang, the degree of automation in post-harvest processing (e.g., precision cutting, grading, drying) can be equally, if not more, distinctive. Yet local producers often note: “The machines are good, but profits go to southern distributors who excel at packaging, decoration, promotion, and branding.” This contrast highlights a perspective central to the GCPM framework: cultivation refinement (‘Automation’ in the GCPM) is a necessary but insufficient condition for economic success. Sustainable advantage is secured only when it is coupled with and enhanced by dominance in the Processing and Market domains, specifically through branding, packaging, propaganda, logistics, and channel control [38,39,40]. The southeastern ecosystem thrives precisely because it often integrates these downstream functions locally, enabling producers to capture a greater share of the final consumer value.

Therefore, the southeastern system represents more than a technological high point; it illustrates how proximate market pressure catalyzes a holistic transition from cost-competitive production to quality-differentiated, value-added agribusiness. It offers a critical policy and development insight: investments in production technology could be strategically coupled with parallel investments in market linkage, brand building, and value-chain governance to ensure that the rewards of precision are accrued by the primary producers themselves, not captured by intermediaries elsewhere [41].

### 3.4. Southwest Transitional Zones: Integrated Cultivation and Ecological Value Addition

The complex, humid, and topographically diverse landscapes of southwestern China (e.g., Guizhou, Sichuan, Yunnan) have fostered cultivation models that exemplify strategic adaptation under constraints. These regions do not have the capital intensity of southeastern factories, nor the vast monocultural resource bases of the north. Instead, they have built resilience through intelligent ecological integration and value innovation. This can be observed in two distinct yet complementary paradigms: circular agro-innovation in basins and distinctive forest symbiosis in highlands.

In the fertile yet land-constrained Sichuan Basin, adaptation focuses on maximizing resource synergy within the agricultural landscape. The large-scale incorporation of mulberry branch chips (30–39%), a by-product of the region’s historic sericulture industry, into the substrate is a signature practice. This move, documented in local protocols [42], could turn a waste stream into a primary input, embodying a circular economy logic that reduces costs and enhances holistic sustainability. Other local agricultural residues are similarly valorized, with formulations and moisture content (62–65%) carefully calibrated for the humid environment.

Simultaneously, in the forested highlands of Guizhou and Yunnan, a deliberately ecologically nuanced understory cultivation model has been refined. This model is an archetypal agroforestry system, as codified in local standards [43]. This system represents a modern articulation of longstanding indigenous agroforestry knowledge, where the forest canopy (density 0.6–0.8) serves as natural infrastructure for microclimate regulation. Practitioners report that this approach reduces energy costs and buffers against temperature extremes. The forest canopy moderates summer highs by 3–5 °C and reduces winter heat loss, while also maintaining higher humidity than open fields. However, direct comparative data with conventional greenhouse systems remain limited. From a resilience perspective, such systems are hypothesized to offer advantages—a proposition that warrants systematic investigation. This practice does more than reduce energy costs; it actively shapes a unique product phenotype. The dappled light, specific microbiota, and microclimate of the forest floor contribute to fruiting bodies with a denser texture and a distinctive, prized woodland aroma. This forms an effective Germplasm–Cultivation–Market linkage. A specific, place-based cultivation practice contributes to a sensory trait (related to processing potential). This trait is highly valued by discerning, niche markets that seek authentic and storied foods.

Thus, the southwestern model could be viewed as presenting an alternative development pathway that emphasizes ecological harmony, cultural distinctiveness, and product uniqueness rather than competing solely on cost-volume efficiency. It indicates how regions can leverage ecological and cultural assets, namely their “agricultural heritage”, to bypass capital-intensive stages of development and aim for high-margin, differentiated market segments [44]. However, this pathway’s success is not automatic. Its key challenges are not primarily technological, but socio-economic. Scalability is limited by the availability of suitable ecosystems. Meanwhile, its economic viability largely depends on the ability to build strong brands, ensure consistent quality, and establish short, trustworthy supply chains. These supply chains connect forest plots to premium urban consumers, helping capture the full value of the “ecological premium.” This model suggests that in an era of market differentiation, strategic integration into a specific ecology can be a more valuable source of competitive advantage than attempting to dominate the environment through industrial means [45,46,47]. It is important to note, however, that the resilience benefits of such understory cultivation systems remain qualitatively described rather than quantitatively demonstrated. While local practitioners and technical standards suggest improved stability and reduced input dependency, comparative studies measuring yield variability, stress tolerance, or economic risk against conventional greenhouse systems have not yet been conducted for these specific contexts. This represents a critical gap in the evidence base. Future research should prioritize addressing this gap. The descriptive accounts presented here provide a foundation for such comparative work by documenting how these systems operate and what variables may matter.

### 3.5. Plateau Climate Zones: Niche Exploitation and the Sanctuary of Genetic Diversity

Cultivation on the high plateaus, such as in Tibet and parts of Yunnan and Sichuan above 2500 m, represents a paradigm of turning existential constraints into multifaceted value. The challenges are severe: chronic hypoxia (oxygen levels ~60% of sea level), intense UV radiation (30–40% higher than lowland areas), and drastic diurnal temperature swings (often 25 °C difference between day and night). These extreme conditions have selected for unique genetic traits in local strains and call for specialized greenhouse designs that can buffer against both cold nights and intense daytime solar radiation. Beyond immediate commercial adaptation, these extreme conditions serve as an ecological filter. They may turn such regions into a sanctuary and diversity hotspot for the *Hericium* genus, including wild relatives like *H. coralloides*, *H. alpestre*, and *H. abietis*. Production is therefore based on two components: the use of locally adapted cultivars and the in situ conservation of wild genetic diversity.

The managed production component represents a significant bio-engineering endeavor, codified in standards [48]. It mandates protected cultivation in energy-efficient, solar-optimized greenhouses, which are designed to buffer extreme temperature swings. Every parameter is fine-tuned for the plateau. For example, sterilization accounts for lower boiling points, substrate formulations make use of available materials, and fruiting is managed within a narrow 15–20 °C window. This system exploits a key phenological advantage: producing summer crops when lowland regions are too hot, creating a valuable off-season market niche. This off-season production strategy differs from the subtropical model. While subtropical zones leverage proximity to urban markets for fresh, high-value sales, plateau cultivation relies on temporal rather than spatial market advantages. The same climate that limits year-round production, with cold winters, also creates a market opportunity when lowland regions become too hot for summer cultivation. This may be seen as one form of climate resilience, not only through withstanding stress but also through temporal niche differentiation.

However, the true strategic significance of the plateau may extend beyond this commercial niche. The populations of cultivated and, more importantly, wild *Hericium* species persisting there constitute a living library of genetic traits forged under extreme stress. These traits include hypoxia tolerance, UV resilience, and metabolic adaptation to cold. They are an invaluable resource for global fungal breeding programs confronting climate change. This natural reservoir of genetic diversity encompasses not only *H. erinaceus* but also related species like *H. coralloides*, *H. alpestre*, and *H. abietis*. It represents a critical resource for future biotechnological applications, from stress-tolerant cultivar development to the discovery of novel bioactive compounds. Thus, the plateau’s role transitions from a mere producer to a guardian of adaptive genetic diversity [49].

This perspective reframes the model’s vulnerabilities. While its economic niche is sensitive to market competition, its value as a genetic sanctuary and a natural laboratory for studying climate adaptation is enduring and potentially growing. The future resilience of the *H. erinaceus* industry globally may well depend on the conservation of such “edge populations” in extreme environments. Therefore, the plateau model offers an important alternative development logic. It may illustrate how regions can leverage their unique extreme ecology, not only for direct production but also as stewards of global agricultural biodiversity. This secures a different form of vital value and provides a possible rationale for targeted conservation and research investment [50]. This aligns with a broader principle: in an uncertain climatic future, preserving the capacity to adapt (genetic diversity) may be as crucial as optimizing for current production [51].

### 3.6. Synthesis: From Chinese Practice to a Conceptual Climate-Adaptation Framework for Global Reference

This scoping review synthesizes regional cultivation practices rather than providing high-evidence conclusions. Given the niche nature of *H. erinaceus* research (English literature <50 papers/year; Chinese technical standards dominate), evidence strength is limited to case reports, expert consensus, and local protocols. Key parameters (e.g., bag thickness 0.005–0.08 mm, segment length 15–30 cm) are derived from verified field practices (Figure 1), not randomized trials. This mirrors the field’s current reality: adaptation relies on localized, experience-based knowledge rather than universal protocols. This reliance on practice-based evidence is viewed as the source of the review’s practical relevance.

The comparative analysis across China’s five agro-climatic zones suggests patterns that extend beyond a catalogue of techniques. It points to the potential utility of a hierarchical, adaptable methodology for designing climate-resilient agricultural systems, which can be described as a three-tiered framework:

1. Germplasm-Level: Directed Domestication under Pressure. The starting point is the targeted selection and domestication of varieties for core environmental stressors (e.g., extreme cold, hypoxia, or thermotolerance). This creates a tailored biological foundation.
2. Cultivation-Level: Ecological Niche Construction with Local Capital. Systems are built by engineering growth media and microclimates from locally abundant, often underutilized resources (e.g., sawdust, crop residues, forest canopy), turning constraints into circular economy advantages.
3. System-Level: Spatiotemporal Optimization of Climate Endowments. The entire production calendar and infrastructure are configured to exploit comparative climatic advantages (e.g., cool summers for counter-season supply, distinct seasons for staggered production).

The five regional systems illustrate the application of this framework, each emphasizing different tiers and thereby confronting distinct trade-offs: the cold temperate system prioritizes energy conservation but risks resource dependency; the subtropical system focuses on biological risk mitigation via technology but faces capital intensity; while temperate, southwestern, and plateau systems blend flexibility, ecological integration, and temporal niche exploitation. Collectively, they illustrate that resilience is not a single prescription but a spectrum of context-specific strategies. The explicit mapping of these strategies and their inherent trade-offs (e.g., dependency, volatility, lock-in) provides a conceptual reference for contemplating resilient system design in other regions.

Taken together, this approach suggests that successful industrialization does not depend solely on standardized, high-input greenhouse models. Instead, it can be a process of strategic localization and intelligent integration, offering a valuable reference for developing context-sensitive, sustainable, and commercially viable cultivation systems for high-value fungi and potentially other crops worldwide. This climate-adapted cultivation regime, integrated with agroforestry and mixed farming, is associated with a dual advantage of ecological resilience and reliable supply for food and nutraceutical industries.

## 4. Emerging Innovations and Inherent Trade-offs: A GCPM Perspective

The ongoing modernization of *H. erinaceus* cultivation is being influenced by a suite of innovations that align with broader global trends in digital agriculture, circular bioeconomy, and advanced breeding. A critical appraisal of these frontiers through the GCPM Integration lens is essential to gauge their far-reaching potential, limitations, and the new dependencies they might create.

### 4.1. Digital Intelligence in Data-Driven Cultivation

The integration of smart agriculture technologies, including the Internet of Things (IoT), artificial intelligence (AI), and digital twins, represents a potential future direction for *H. erinaceus* cultivation. Such technologies could move cultivation beyond the empirical, reactive management described in region-specific protocols towards more proactive, predictive approaches, though their application to *H. erinaceus* remains exploratory. Crucially, the application and value of these technologies are not uniform. They should be carefully evaluated and tailored to address the distinct agronomic challenges, infrastructure conditions, and socio-economic contexts of each climatic zone.

In the Cold Temperate zones (e.g., Hailin), where the paramount challenge is thermal efficiency and energy conservation against prolonged winters, data-driven solutions offer potential precise optimization. IoT sensor networks can be deployed within the semi-underground, insulated mushroom houses to monitor real-time temperature gradients, substrate core temperature, and humidity levels. This granular data stream can feed AI algorithms designed to dynamically manage heating systems (e.g., underfloor heating, radiators). By integrating external weather forecasts, these systems can pre-emptively adjust energy inputs to maintain the critical, narrow fruiting window of 14–18 °C, minimizing fuel or electricity consumption while stabilizing the microclimate. This intelligent thermal management addresses the system’s core vulnerability, namely its dependency on intensive, localized energy inputs, by enhancing resource-use efficiency. Here, digital intelligence serves to fortify a specialized, input-dependent system against its primary economic and environmental pressure point.

Conversely, in the industrial-scale Subtropical systems (e.g., Gutian, Changshan), digital tools are deployed to manage the ever-present bioclimatic risks of high temperature and humidity. Digital twins, virtual replicas of greenhouse environments, have been explored in other agricultural contexts to model complex physical processes such as airflow, heat exchange, and moisture distribution [52,53,54]. Similar approaches could eventually be adapted for *H. erinaceus* cultivation, though no documented applications currently exist. This application of digital twins for microclimate optimization aligns with their growing use in controlled environment agriculture to model plant growth and resource dynamics [52,55,56]. In principle, such models could be used to simulate different environmental scenarios and optimize the operation of climate control systems, potentially helping to disrupt conditions that favor contamination by Trichoderma spp. and other pests. Furthermore, computer vision systems powered by AI can perform continuous, non-invasive monitoring of mycelial growth and fruiting body development. Early detection of morphological anomalies or disease spots could enable targeted, timely interventions, reducing reliance on broad-spectrum prophylactic measures, though the efficacy of such targeted interventions may vary by strain and contamination pressure. In this context, digital intelligence supports the region’s shift toward capital-intensive precision. It helps move from scheduled automation to adaptive, risk-aware management. This can help safeguard high-value yields for premium markets.

Looking further ahead, digital tools might also support understory cultivation systems. The Southwest Transitional and understory cultivation models present a different opportunity for digital augmentation. Here, the cultivation practice is deeply embedded in a natural ecosystem, relying on experiential knowledge to leverage the forest microclimate. Low-cost, wireless IoT sensor networks may expand access to crucial data, provided that local digital infrastructure and operational capacity are available. Sensors measuring photosynthetically active radiation (PAR) under the canopy, soil moisture, and ambient humidity across dispersed forest plots can provide objective metrics to guide decisions on bag placement, irrigation scheduling, and optimal harvest windows. This technology does not aim to replace the system’s ecological wisdom, but to augment it. It complements rather than overrides place-based experiential knowledge, helping translate tacit knowledge into scalable, data-informed practices. For these value-added, niche-market-oriented systems, digital tools enhance consistency, traceability, and management efficacy, potentially bolstering the “ecological premium” strategy. The verifiable data they could generate on growing conditions could become a valuable asset for branding and market storytelling.

However, this promising digital transition is fraught with inherent trade-offs that are acutely visible through the lens of regional disparity. Advanced AI and digital twin systems require high capital investment, technical expertise, and digital infrastructure. These requirements may exacerbate a “precision divide”. Notably, this divide is not inevitable but can be mitigated through policy support for smallholder cooperatives and public–private partnerships to share infrastructure costs [57]. The risk of a ‘precision divide’ mirrors well-documented concerns in the broader literature on agricultural technology adoption, where benefits often accrue disproportionately to larger, resource-rich producers [58]. This divide may further strengthen the advantages of large, well-capitalized entities in regions such as the industrial Subtropical zones. At the same time, it could marginalize smallholder producers and community-based operations in the Southwest Transitional, Plateau, and even Temperate zones. Such a trajectory could deepen existing inequalities in market access, value capture, and resilience-building capacity, thereby undermining the social sustainability integral to a truly intelligent and integrated agricultural system. The environmental calculus is also complex [59,60]. While operational resource efficiency gains are substantial in targeted scenarios, they should be weighed against the lifecycle environmental costs of manufacturing, operating, and disposing of electronic hardware and data centers, which tends to scale with the intensity of digital deployment. A comprehensive sustainability assessment should therefore consider the embodied energy and e-waste, issues highlighted in life cycle assessments of ICT for sustainable development [61,62,63].

Therefore, achieving an equitable and sustainable digital future for *H. erinaceus* cultivation requires context-sensitive pathways. This includes developing inclusive business models (e.g., cloud-based data analytics or sensor leasing for cooperatives), fostering participatory design of user-friendly interfaces tailored to local literacy levels, and implementing targeted policy arrangements that support digital skill development and infrastructure sharing. Participatory design and tailored business models, such as ‘Technology-as-a-Service’ models, have been advocated in development studies to enhance the relevance and accessibility of innovations for diverse user groups [64]. The goal is to ensure that the benefits of data-driven cultivation are accessible across diverse production systems. With appropriate contextual adaptation and intelligent integration, all regions may be able to improve their climate resilience and market competitiveness.

### 4.2. Circular Resource Integration and Valorization

Concurrent with digitalization, the principle of the circular bioeconomy is being operationally embedded within *H. erinaceus* cultivation, evolving from simple substrate substitution towards distinctive, multi-loop resource integration. This paradigm aims to turn linear “take–make–dispose” flows into circular systems. It can address both the resource dependency and sustainability paradoxes observed in the climate-zone models. The implementation, however, varies considerably based on regional resource endowments and system boundaries.

In the Temperate and Subtropical zones, where cultivation is often large-scale and proximate to major crop production, circular integration primarily focuses on the valorization of primary agricultural wastes. Cottonseed hulls, corn cobs, and rice bran are heavily utilized in substrates. This practice illustrates industrial symbiosis, turning potential disposal burdens into valuable inputs [65,66]. The environmental benefit is twofold: reducing the environmental footprint of both the source crop (by finding a use for its residue) and the mushroom (by avoiding dedicated forestry harvests). The stability of this model, however, is closely tied to the continuity and quality of these external waste streams, making the cultivation system vulnerable to shifts in adjacent agricultural markets and practices.

Moving beyond initial substrate formulation, a key circular innovation lies in the management of Spent Mushroom Substrate (SMS). In the centralized, capital-intensive systems of the Subtropical and some Temperate zones, SMS is increasingly seen not as waste but as a feedstock for secondary valorization. Controlled anaerobic digestion of SMS can yield biogas for on-site energy generation, while the resulting digestate serves as a nutrient-rich organic fertilizer or soil amendment. Some integrated production systems explore cascading SMS into insect farming (e.g., as feed for black soldier fly larvae), producing protein for animal feed and frass as a high-quality fertilizer [67,68]. This creates a synergistic waste-substrate-product-energy-fertilizer/feed loop. It can theoretically move toward closed resource cycles and lower overall system emissions. This advanced integration represents a relatively high-efficiency, technology-dependent circular model that aligns with the industrial character of these production hubs.

**Figure 4:**
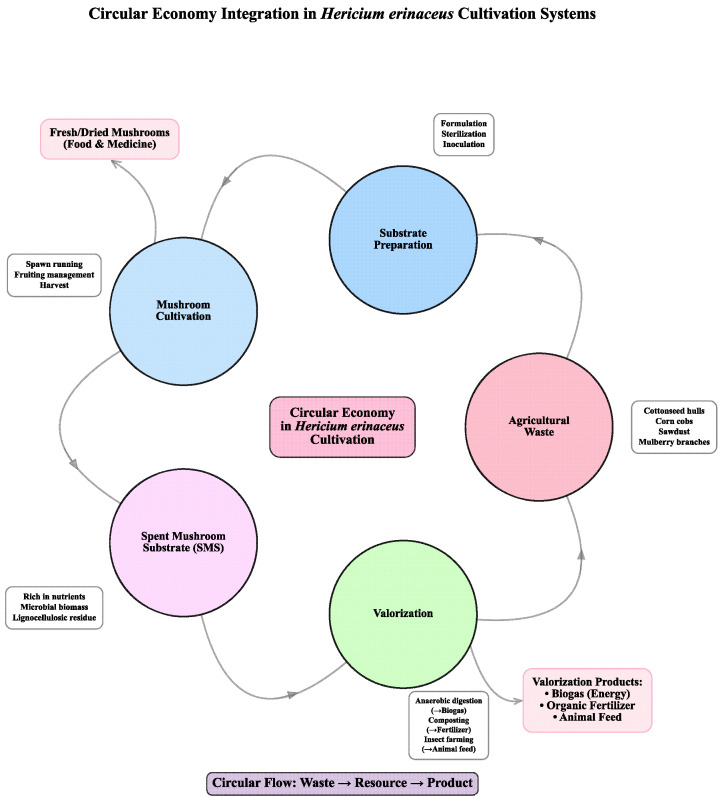
Circular economy integration in *H. erinaceus* cultivation systems.

In the Southwest Transitional and understory systems, circularity manifests differently, rooted in spatial and ecological integration. Local mulberry branches and forest thinning residues are used in the growing region. This reduces transportation emissions and reflects a localized circular economy shown in Figure 4. Here, the “circular” benefit is associated with the cultural and ecological identity of the product given the limited availability of region-specific biomass, enhancing its sustainability narrative for niche markets. However, the scale of such loops is limited by the local availability of these specific biomass streams.

The push towards circularity, while environmentally promising, unveils critical long-term inherent trade-offs that demand rigorous assessment. A primary concern is the risk of contaminant accumulation and biological imbalance in closed-loop systems. Continuous recycling of SMS can occur in several ways: as a substrate component, after digestion, or via insect frass. Such recycling may lead to gradual accumulation of salts, heavy metals (if present in initial feedstocks), persistent fungal pathogens, and autotoxic metabolites. This depends on the initial feedstock quality and recycling frequency [69,70]. This could degrade substrate quality over time, suppress yields, and compromise the safety of downstream products (e.g., fertilizers, feed) particularly in long-term continuous recycling scenarios. Thus, the sustainability of circular models depends on continuous monitoring, the development of effective pre-treatment or purification protocols, and a deep understanding of the biological and chemical dynamics in these recycled matrices. This remains a significant, ongoing research and management challenge.

Furthermore, the economic viability of advanced circular processes like anaerobic digestion is highly sensitive to scale and policy, where economies of scale can be achieved and supportive policies are in place. It often requires substantial initial investment and technical expertise, potentially creating a “circularity divide” analogous to the digital divide, where only large-scale producers can afford to capture these advanced sustainability benefits. To mitigate this, targeted support could include subsidizing small-scale pre-treatment equipment or facilitating cooperative models for aggregating SMS from individual growers, enabling economies of scale in centralized processing. Therefore, promoting circular integration should be combined with strategies to ensure inclusive access to technology. For example, supporting smallholder cooperatives to aggregate SMS for centralized processing and innovating business models. This helps ensure that the transition to a circular bioeconomy does not inadvertently marginalize small-scale producers in less industrialized regions [71,72].

### 4.3. Germplasm Innovation: Expanding Biological and Market Frontiers

Germplasm innovation remains the foundational driver for expanding the ecological and commercial boundaries of *H. erinaceus* cultivation. It shapes opportunities across the GCPM framework, enabling new cultivation geographies, product forms, and market opportunities. The strategic focus of this innovation, however, is shaped by the pressures and opportunities unique to each climatic zone.

In the Cold Temperate and Plateau zones, germplasm innovation is primarily about abiotic stress tolerance. The domestication and breeding of strains like ‘Heiwei 9910’ for sub-zero resilience or the selection of hypoxia-tolerant varieties on the Tibetan Plateau represent a biological solution to existential environmental constraints. These varieties do not merely survive; they are optimized to complete their lifecycle with quality and yield within a narrow, stress-defined window. This could turn a severe limitation into a unique niche, as long as strains are well-adapted to local management practices. It allows these regions to produce at times and places where others cannot.

Beyond cultivated varieties, the wild populations in these extreme environments, including relatives like *H. coralloides* and *H. alpestre*, constitute an invaluable reservoir of genetic diversity [73,74,75]. This “natural library” contains alleles for traits that may become critical for global climate change adaptation. It elevates these regions from producers to guardians of adaptive genetic resources. The importance of such wild genetic resources is underscored by molecular studies of cultivated strains. For example, inter-retrotransposon amplified polymorphism (IRAP) analysis of 34 cultivated *H. erinaceus* strains in China revealed that, while genetic differences exist among strains, the overall genetic correlation is high, suggesting a relatively narrow genetic background among commercial cultivars [76]. This finding points to the potential value of incorporating wild germplasm particularly from extreme environments such as the Plateau into future breeding efforts. Recent advances in genomic research have begun to provide the tools needed for such efforts. The de novo sequencing and assembly of the *H. erinaceus* monokaryon CS-4 genome, using Illumina and PacBio platforms, generated a 41.2 Mb genome encoding 10,620 predicted genes, including 341 carbohydrate-active enzymes involved in lignocellulose degradation and 447 transcription factors [77]. Importantly, this study also characterized genome-wide microsatellites and developed a comprehensive marker database (HeSSRDb) containing 904 microsatellite markers [77]. These genomic resources and molecular markers may enrich the toolbox for biological and genetic studies in *H. erinaceus*, potentially facilitating germplasm characterization and the exploration of genetic relationships relevant to future breeding programs.

In the Subtropical and Temperate industrial belts, germplasm innovation is increasingly driven by market-oriented traits and production efficiency. Here, breeding objectives extend beyond environmental adaptation to include characteristics like rapid, uniform growth cycles, high biological efficiency (yield per substrate), resilience against region-specific pathogens (e.g., thermotolerant strains with enhanced defense against Trichoderma), and important post-harvest qualities (e.g., shelf-life, texture, pale coloration) [78,79]. Furthermore, the deliberate development of strains with elevated profiles of specific bioactive compounds (e.g., Erinacine A for neurocognitive markets, or immunomodulatory polysaccharides) illustrates a Germplasm-to-Market linkage. This can help producers target high-end functional food and nutraceutical markets, which require rigorous safety and efficacy validation [80]. It adds value beyond basic commodity production.

The strategic innovation in the Southwest Transitional zones often lies in leveraging unique species and place-based phenotypes. The cultivation of related species like *H. coralloides* (Coral Tooth Mushroom), with its distinctive appearance and flavor, has opened new gourmet product categories. Similarly, forest-cultivated *H. erinaceus* is suggested by some growers and foragers to develop a denser texture and a more pronounced “woodland” aroma compared to substitute cultivated forms. This observation raises the possibility that, as with wine, coffee, and specialty cheeses [81,82,83,84], interactions between genotype and local environmental conditions, sometimes referred to as terroir, may contribute to the sensory traits of wild or forest-based mushrooms. However, empirical studies linking specific environmental factors (e.g., light spectrum, microbiota, microclimate) to sensory attributes in *Hericium* species remain scarce, and such hypothesized effects await systematic investigation. In these systems, the germplasm (whether a different species or a locally adapted strain) is inseparable from the cultivation practice and the story of its origin. This fusion creates a holistic, non-replicable product identity that may command an ecological premium depending on effective branding and consumer recognition. Such premiums are increasingly captured in markets for geographically indicated (GI) products and agroecologically certified goods, where consumers pay more for authenticity, sustainability narratives, and perceived superior quality linked to specific production practices and origins [85]. The understory cultivation model itself illustrates agroforestry systems designed for multiple benefits, including biodiversity conservation and sustainable livelihoods, which further reinforces its sustainability brand equity [86,87,88].

The contrasting breeding strategies across climatic zones suggest an underlying pattern: germplasm adaptation tends to reflect the nature of the dominant stress. In the cold northeast, where a single, predictable stress (winter cold) dominates, strains have been selected for extreme tolerance to that specific stress. In the temperate zone, where variability itself is the challenge, strains exhibit broad thermal plasticity rather than extreme tolerance. In the subtropics, where multiple stresses (heat, humidity, pathogens) co-occur, strains appear to combine tolerance traits in more complex ways. This gradient from single-trait to multi-trait adaptation resonates with ecological concepts of specialization versus generalization.

The acceleration of these breeding efforts could be further supported by modern tools that have shown promise in other fungal species [89,90,91]. For example, desirable traits might be pyramided through molecular marker-assisted selection, and novel genetic variation could be generated by techniques like ARTP mutagenesis. Advanced genomic and phenomic platforms, increasingly used in fungal research, may eventually allow for the dissection of complex traits in *H. erinaceus*, potentially moving breeding from a slow, phenotypic art towards a faster, more predictive discipline. However, the application of these tools to *H. erinaceus* remains in its early stages.

Despite this progress, the global potential of germplasm innovation is constrained by key cross-cutting barriers in genetic resource integration. One such barrier is limited access to elite germplasm for smallholder producers in resource-poor regions. This creates a paradox: regions with advanced breeding capacity may lack genetic diversity from extreme environments, while regions rich in such diversity (e.g., the Plateau) may lack the capacity to fully characterize and utilize it. This stifles the international collaborative trialing and pre-breeding efforts essential for developing broadly resilient, “climate-smart” varieties, representing a critical failure in global system integration [92,93].

Furthermore, the focus on developing proprietary, high-performing hybrids for intensive systems risks accelerating genetic uniformity in large-scale commercial production regions, potentially increasing overall vulnerability to emerging pests or climate shifts, which represents a classic trade-off between productivity and resilience. Therefore, a balanced germplasm strategy should combine elite cultivar development with in situ and ex situ conservation. Examples include community seed banks for traditional strains and collaborative networks for sharing wild and traditional genetic diversity. This helps maintain a broad genetic base for future adaptation [94]. This suggests that true “intelligent integration” in germplasm may require not only advanced science but also equitable international cooperation and robust policies governing genetic resource stewardship [95].

From wild relatives to regionally adapted cultivars, China’s *H. erinaceus* germplasm diversity supports current breeding for climate resilience, provides genetic resources for improved product quality, and enables development of region-specific products with distinct sensory traits. This review documents where different strains are currently used and under what conditions, offering a practical reference for future research on characterizing and utilizing this diversity through breeding or conservation efforts. This germplasm diversity is closely linked to the diversity of cultivation systems across China’s climatic zones, as each system has selected for strains best suited to local conditions.

### 4.4. Bioactive Compound Valorization: Bridging Cultivation and Nutraceuticals

Bioactive compounds from *H. erinaceus* are important drivers for its food and nutraceutical value, with cultivation parameters influencing their yield and bioavailability. Beyond innovations in cultivation technology, the frontier of bioactive compound discovery and valorization is substantially reshaping the economic potential and market structure of the *H. erinaceus* industry. This domain reflects the core logic of the GCPM framework. It establishes a clear value-driven link: cultivation parameters are optimized to improve specific processing outcomes (extract yield and compound profile). These outcomes then define product efficacy and market positioning (nutraceuticals, functional foods). The pursuit of this valorization pathway, however, interacts distinctly with the resource bases and climatic pressures of different production zones.

The foundational step is the cultivation-phase modulation of bioactive compound biosynthesis. Research shows that production of key metabolites such as HEPs and Erinacines is not static. It is highly responsive to abiotic stresses and substrate composition, representing a clear G × E × M interaction [96]. For instance, the synthesis of defensive secondary metabolites may be upregulated by controlled mild drought stress though the magnitude of this effect varies by strain and stress intensity or temperature fluctuations during specific growth stages—strategies feasible in the Temperate zones [97,98]. Similarly, the unique spectral quality of dappled light in Southwest Transitional forest understories, or the intense UV exposure on the Plateau, could influence the metabolic pathways yielding these compounds. This aligns with the broader principle in plant and fungal science that environmental cues can be harnessed to steer metabolism towards desired phytochemical profiles, a concept central to the production of high-value botanicals and medicinal plants [99,100,101].

Post-harvest, the challenge shifts to efficient extraction, stabilization, and formulation to create bioavailable and market-ready products. Emerging research on bioactive peptides like KSPLY illustrates this transition from raw biomass to refined ingredient [8]. The efficacy of such peptides in modulating immune responses or gut microbiota indicates their potential as targeted nutraceuticals subject to further clinical validation and regulatory approval. However, moving from lab-scale discovery to commercial production involves key Processing hurdles. The extraction efficiency, stability during processing, and vital oral bioavailability of these compounds are critical determinants of commercial viability [102]. For example, health claims for bioactive peptides or polysaccharides often require extensive clinical data to meet regulatory thresholds, which can be time-consuming and costly for producers. Techniques such as enzymatic hydrolysis, fermentation-assisted extraction, or microencapsulation may be needed to improve stability. These require significant technological investment, which can be a barrier for small-to-medium enterprises and regions with limited capital. This creates a potential divergence: regions with advanced Processing infrastructure (e.g., proximate to biotech hubs in Subtropical coastal zones) may be better positioned to capture this high-value segment, highlighting the need for targeted support to build processing capacity in resource-rich but infrastructure-poor regions, whereas regions focused on fresh or dried commodity production may remain suppliers of raw material.

This valorization pathway intensifies the Germplasm–Market linkage. It incentivizes the breeding or selection of strains not just for agronomic traits, but as “chemical factories” optimized for high titers of specific HEP fractions, Erinacine A, or peptides. This illustrates a typical example of market-pull breeding [103,104,105]. Consequently, the Plateau’s wild germplasm, evolved under extreme stress, could be seen as a particularly valuable resource for discovering novel chemical scaffolds or high-potency alleles.

However, this focus on isolated compounds introduces new inherent trade-offs and vulnerabilities. First, it may prioritize chemical profile over other quality attributes (e.g., taste, texture for the fresh market), potentially creating two divergent product streams and market channels. Second, the industry risks “compound-centric” reductionism, where the synergistic effects of the whole mushroom or crude extract (the “entourage effect”), which may enhance bioavailability or reduce side effects, are undervalued in favor of single, patented molecules [106,107,108]. This could alter supply chains, intellectual property landscapes, and health claims. Finally, the economic sustainability of investing in extraction and purification technology depends on robust, science-backed health claims and regulatory approval, which is a non-trivial barrier tying the sector’s fate to the evolving and often stringent global regulations, such as those governing health claims, safety assessment, and product labeling in major markets for nutraceuticals and dietary supplements [109,110].

In summary, bioactive compound valorization represents both a major opportunity for upgrading the *H. erinaceus* value chain and a force that could reshape its geographic and economic contours. It requires closer, data-driven integration across the GCPM framework. This includes alignment with regulatory requirements and consumer safety expectations, from germplasm selection and cultivation stress management to advanced bioprocessing. All steps should be guided by market intelligence on health trends and regulatory pathways.

## 5. Towards Predictive Cultivation: An Integrated Roadmap for Climate Resilience and System Intelligence

A progressive trajectory for *H. erinaceus* cultivation can be derived from synthesizing climate-zone-adapted systems and critically evaluating key innovations. These include digital intelligence, circular resource integration, germplasm improvement, and bioactive compound valorization. They suggest an evolution from localized, experience-based practices towards increasingly systematic and technology-augmented paradigms depending on regional technical capacity and resource availability. However, to fully address the climate vulnerabilities, resource dependencies, and sustainability paradoxes embedded in current models, a gradual, context-sensitive paradigm shift is required: from optimizing individual components in isolation to architecting integrated, climate-resilient, and intelligently managed cultivation ecosystems [111]. A dual-pillar roadmap to operationalize this shift is outlined, grounded in and extending the GCPM framework and tailored to regional resource endowments and socio-economic capacities. Pillar One focuses on building dynamic, climate-resilient systems through intelligent, cross-node integration. Pillar Two targets major scientific knowledge gaps. These include G × E × M interactions, long-term contamination risks in circular systems, and bioactive compound bioavailability under field conditions. Closing these gaps will enable a shift from empirical adaptation to predictive design, with priority on context-relevant research that addresses region-specific vulnerabilities.

### 5.1. Pillar One: Building Climate-Resilient Systems Through Intelligent Integration

The current climatic zone models, while distinctive, represent a degree of static optimization for historical climate norms. However, their robustness in the face of accelerating, non-linear climate change remains uncertain. This climate change is characterized by more frequent and intense extreme weather events, shifting precipitation patterns, and rising baseline temperatures. Building dynamic resilience is therefore a key priority and can be achieved through the feedback-driven, synergistic approach that the GCPM framework seeks to capture. Resilience should be engineered simultaneously across all nodes: in Germplasm through adaptive genetic traits, in Cultivation through predictive and adaptive management, in Processing through flexible and cascading valorization pathways, and in Market through diversified and robust linkages that can absorb shocks.

Predictive Germplasm Development for a Dynamic Climate: Looking forward, breeding programs may need to transcend the selection for static tolerance thresholds (e.g., survival at 35 °C) and instead prioritize the genetic architecture of phenotypic plasticity and yield/quality stability across variable and combined stresses (e.g., heat + drought, hypoxia + UV) [112,113,114]. This goal could eventually be supported by establishing international, high-throughput phenotyping platforms. These platforms can screen global germplasm collections, including valuable wild relatives and landraces conserved in extreme environments like the Plateau, under simulated future climate scenarios in controlled environments. If coupled with advanced genomic (e.g., whole-genome sequencing, GWAS) and transcriptomic tools, such platforms could support the development of “climate-smart” or “intelligent germplasm”. While these approaches have been explored in model organisms and some crops, their application to *H. erinaceus* breeding remains a future prospect that would require sustained research investment and regional capacity building [115].

Dynamic, Data-Driven Cultivation Management: To operationalize the potential of advanced germplasm, static, calendar-based protocols should be gradually supplemented and optimized by adaptive, predictive management regimes. This involves creating decision-support systems (DSS) that integrate sub-seasonal to seasonal (S2S) climate forecasts with biophysical crop models specifically developed and calibrated for *H. erinaceus* [116,117]. Such a DSS, fed by real-time IoT sensor data from the cultivation environment and substrate, could provide actionable intelligence. Scaled-down versions can be tailored for smallholder producers and resource-limited regions, offering specific guidance: advising on optimal spawning dates to avoid predicted heatwaves during the critical primordia initiation phase, recommending precision irrigation schedules based on forecasted evapotranspiration and substrate moisture data, or suggesting alternative, locally available substrate recipes when conventional input supply chains are affected by drought. This could turn the Cultivation node from a reactive, schedule-follower to an intelligent, proactive manager.

Systemic resilience should extend beyond the biological production phase to encompass the physical and economic infrastructure of the value chain. Physical cultivation infrastructure (e.g., greenhouses, mushroom houses) may benefit from re-evaluation and redesign based on regional climate characteristics and economic capacity for passive climate buffering, incorporating principles of energy efficiency, renewable energy integration (such as photovoltaic greenhouses), and water harvesting. For example, cold temperate zones may prioritize insulated, energy-efficient mushroom houses to reduce winter heating costs, while subtropical zones focus on ventilation and cooling systems to mitigate high-temperature stress. Concurrently, market structures and supply chains should build in redundancy, flexibility, and value-added pathways. This includes diversifying sourcing networks across geographically dispersed climate zones to buffer against regional climate shocks—a strategy that leverages the considerable diversity of China’s production models [118,119,120]. Furthermore, developing robust secondary processing streams (e.g., for extraction, canning, drying) can help ensure marketability and reduce waste when fresh-market quality is compromised by abiotic stress. Low-cost, scalable technologies accessible to small-to-medium enterprises would be essential for such efforts, thereby enhancing the overall resilience of the market-linked system. Financial de-risking instruments, such as index-based insurance tailored to mycoculture, can further stabilize producer livelihoods and ensure the conversion of climate-resilient harvests into stable food and nutraceutical products. Additionally, capacity-building programs for smallholders, including training on simplified DSS operation and low-cost infrastructure upgrades, can enhance their ability to participate in these resilient systems.

### 5.2. Pillar Two: Deciphering Substantial Interactions for Predictive Integration

The current knowledge base, richly detailed in empirical technical protocols, is robust in the ‘how-to’ but comparatively weak in the mechanistic ‘why’. To achieve the predictive, model-driven integration outlined in Pillar One, there is a need to decode key biological, chemical, and physical interactions among Genotype (G), Environment (E), and Management (M). These G × E × M interactions largely determine phenotype, including yield, quality, and bioactive compound profiles that shape processing potential and market value. The climate-zone-specific systems analyzed are leveraged as a series of invaluable “natural experiments” each providing context-specific data to decipher regionally distinct G × E × M interactions, providing the real-world, multivariate data necessary to advance from descriptive ethnobotany to predictive systems science. Closing this knowledge gap is a key to transitioning from trial-and-error optimization to first-principles design.

Elucidating the Molecular Basis of G × E × M: Critical questions abound at the molecular level that sit at the heart of the Germplasm–Cultivation–Processing continuum with answers likely varying by strain and climatic zone [121,122,123]. How does the transcriptome and metabolome of a cold-adapted strain (e.g., ‘Heiwei 9910’) rewire its chaperone networks and membrane lipid metabolism under short-term heat stress, and how to shift metabolic flux away from the synthesis of valued secondary metabolites? How do specific light spectra and photoperiods, a key Cultivation management tool, precisely modulate the expression of genes encoding polysaccharide synthases or polyketide synthases, thereby linking an environmental cue to the biosynthesis of HEPs and other bioactives valued by the Processing and Market nodes? Deciphering these mechanisms will help to provide the substantial, modular understanding required for predictive models.

Beyond the sterilized, simplified microbiome of artificial cultivation bags, the complex microbial consortia in wild or forest understory environments represent a critical, relatively untapped knowledge frontier. In these natural settings, *H. erinaceus* does not grow in isolation but is embedded in a dynamic web of cross-kingdom interactions with bacteria, archaea, and other fungi inhabiting the soil, decaying wood, and rhizosphere. This ecological microbiome constitutes a substantial component of the natural “Environment” (E) that co-shapes the phenotypic expression of the fungal “Genotype” (G). There is a need to investigate these complex microbial assemblies. Understanding how they are shaped by forest type, climate, and soil chemistry, and how they influence mycelial fitness, nutrient uptake, defense signaling, and bioactive compound profiles, will be important for advancing this field. Deciphering these natural G × E × M × Microbiome interactions provides a crucial reference model [124,125,126]. However, deciphering these multi-partite interactions is inherently complex, requiring advanced metagenomic, metabolomic, and bioinformatic tools, as well as long-term field monitoring to disentangle the roles of individual microbial taxa. It can suggest synergistic microbial partnerships or protective functions lost in sterilized cultivation. This knowledge can then be potentially reverse-engineered to inform the design of next-generation “probiotic” substrate formulations following rigorous in vitro and field validation or synthetic microbial communities for managed cultivation, effectively transplanting ecological resilience from the forest floor into the production system.

Developing in silico predictive models for system design: The key goal of this scientific decoding is integration. The aim is to synthesize data from genetic profiles, real-time and forecasted environmental data, management inputs, and microbiome states into powerful, mechanistic in silico models or digital twins [127,128,129,130]. As data availability and model calibration improve, these models can gradually simulate and predict outcomes for target locations, climate scenarios, or specific production objectives (e.g., maximizing yield, increasing Erinacine A content, optimizing fresh-market appearance). This represents a potential future direction of the GCPM framework, subject to sustained interdisciplinary research and data integration efforts: the ability to design and manage cultivation systems from first principles, allowing for multi-objective optimization (productivity, quality, sustainability, resilience) and dynamic scenario planning. It would enable a predictive science for cultivation, in which management decisions are guided by simulated outcomes prior to their application in the field. Initially, these models could be developed for high-priority production zones (e.g., subtropical industrial belts, plateau germplasm hotspots) to prioritize data collection and calibration, with gradual expansion to other climatic zones as research advances.

### 5.3. Conclusion: From Empirical Practice to Predictive Design Science

Notably, this scoping review focuses on Chinese cultivation systems and does not comprehensively cover global practices, which represents a key limitation that future reviews could address. We have synthesized the climate-zone-adapted cultivation systems of *H. erinaceus* in China and critically appraised emerging innovations through the lens of an integrated framework. The integrated roadmap proposed here offers a perspective. The future of *H. erinaceus* cultivation, and climate-resilient specialty crop agriculture more broadly, may not hinge on perfecting any single innovation in isolation, but could benefit from their intelligent integration guided by a framework such as GCPM. This integration is two-fold: it works horizontally—across the germplasm–cultivation–processing–market continuum to build synergistic, system-level resilience—and vertically, from empirical observation down to molecular and ecological mechanisms to enable genuine predictive power.

The GCPM framework offers a potentially useful lens for examining agroforestry and mixed farming systems for high-value fungal cultivation, particularly for researchers interested in assessing their potential contributions to climate resilience. By highlighting interconnections across the value chain, the framework can help identify where resilience might be built, or where vulnerabilities may lie, in such diversified systems. It serves as an analytical lens to diagnose disconnections and vulnerabilities in current systems. More importantly, it outlines a conceptual design approach for developing next-generation climate-resilient, sustainable, and equitable cultivation ecosystems, pending further validation across diverse contexts. Realizing this vision requires a concerted, transdisciplinary effort: prioritizing research to close the critical G × E × M knowledge gaps; fostering inclusive business, data-governance, and technology-access models to prevent a ‘precision divide’ or ‘circularity divide’; and rigorously validating the environmental and social performance of different integrated pathways through comprehensive life-cycle and systems-level assessments.

Direct comparative studies are needed. These studies should test whether agroforestry-integrated systems enhance climate resilience. They require standardized metrics and long-term monitoring to produce robust and replicable results. Such studies should collect quantitative data on key metrics from paired systems—for example, understory cultivation versus conventional greenhouses—in the same climatic and market contexts. The IPCC Sixth Assessment Report highlights the importance of this research. It identifies ecosystem-based approaches, including agroforestry, diversification and land restoration, as effective adaptation options. These approaches can support food production while providing multiple benefits, such as yield stability and ecosystem health [131]. The Agriculture, Forestry and Other Land Use (AFOLU) sector offers significant mitigation opportunities. It can deliver food, wood and biodiversity conservation, provided the sector adapts to climate change [132]. China’s understory cultivation models, as documented in this review, illustrate possible examples of such ecosystem-based adaptation. These models may serve as references for future IPCC assessments and could provide useful starting points for countries considering the integration of fungal agroforestry into their climate strategies.

By implementing this agenda, the global community can gradually advance *H. erinaceus* cultivation from localized, experience-based practices (however distinctive) toward a predictive design science, and from a commodity-focused activity toward a more equitable and ecologically integrated component of sustainable food and bioeconomy systems. We suggest that climate resilience in *H. erinaceus* cultivation stems not from fortifying any single link, but from fostering a self-reinforcing integration across the continuum. By documenting China’s region-specific cultivation practices and the diversity they embody, this review seeks to lay a foundation for future work that harnesses *Hericium* diversity. This diversity spans across species, locally adapted cultivars, and production systems. The practices themselves, from forest understory cultivation to industrial-scale production, represent a form of applied diversity that can inform climate-resilient agriculture and the development of region-specific functional food products. The Chinese experience synthesized in this scoping review offers a set of adaptation strategies. Through the GCPM framework, it also seeks to offer a conceptual reference for this important transition in modern agriculture, especially for regions with similar agro-climatic and socio-economic conditions. These adaptation strategies are not intended to be replicated, but rather to inspire region-specific adaptations that align with local resource endowments and socio-economic capacities. This framework and approach provide a contextually relevant reference for designing climate-resilient agroforestry and mixed farming systems with particular relevance for regions facing similar climatic transitions and high-value fungal cultivation needs, with particular insights for regions like Europe facing similar climatic transitions.

## Figures and Tables

**Table 1:** Synthesis of key agronomic parameters from Chinese local technical reports on *H. erinaceus* cultivation.

Technical Report (Source)	Region & Climate Zone	Cultivation Model	Key Substrate Formula (s)	Sterilization Key Parameters	Spawn Running Temp. (°C)	Fruiting Management Key Parameters	Primary Pest/Disease Control Strategy
Hailin Municipal Administration for Market Regulation, 2020	Hailin, Heilongjiang (Cold Temperate)	Facility-based (Mushroom House)	1. Spring Formula: Sawdust 81%, Wheat bran 15%, Corn meal 3% 2. Autumn Formula: Sawdust 83%, Wheat bran 15%, Corn meal 1%	High-pressure: 125 °C, 3 h Atmospheric: 100 °C, 8 h + 8 h steaming	18–25	Temp.: 18–22 °C RH: 60–85% Light: 100–200 Lux Key Practice: Two-stage spawn running; semi-underground, insulated houses.	Emphasis on agricultural & physical methods. Chemical control as supplement only; no pesticides during fruiting.
Fujian Provincial Department of Agriculture and Rural Affairs, 2023	Fujian (Subtropical)	Seasonal & Factory-based	1. Cottonseed Hull-based: Cottonseed hulls 76%, Sawdust 10%, Wheat bran 10%, Corn meal 2% 2. Sawdust-based: Sawdust 78%, Wheat bran 20%, Gypsum 1%	High-pressure: 121 °C, 0.10 MPa, 1.5–2 h Atmospheric: 100 °C, 24 h	22–26	Temp.: Primordia 12–18 °C, Fruit body 15–20 °C RH: Primordia 80–85%, Fruit body 85–95% CO_2_: <0.08% Light: 200–600 Lux Key Practice: IoT sensors; automated ventilation & cooling.	Integrated management. Chemicals comply with NY/T 393; emphasis on environmental control.
Zhejiang Provincial Department of Agriculture and Rural Affairs, 2023	Zhejiang (Subtropical)	Green Production, Facility-based	1. High Cottonseed Hull: Cottonseed hulls 70%, Sawdust 15%, Wheat bran 14% 2. Mixed Substrate: Cottonseed hulls 39%, Sawdust 39%, Corn cobs 8%, Wheat bran 13%	Atmospheric: 100 °C (reach in 4 h, maintain 16–18 h)	20–24	Temp.: 14–18 °C RH: Induction 80–85%, Fruit body 85–90% CO_2_: <0.07% Light: 200–400 Lux Key Practice: Standardized workflow; resource efficiency focus.	“Prevention first, comprehensive control”. Priority to non-chemical methods.
Qiandongnan Prefecture Market Supervision Administration, 2022	Qiandongnan, Guizhou (Southwest Transitional)	Understory (Pine Forest)	1. Complex Formula: Sawdust 60%, Cottonseed hulls 20%, Wheat bran 15%, Rice bran 3%, Calcium superphosphate 1%, Gypsum 1% 2. Simple Formula: Sawdust 78%, Wheat bran 20%, Gypsum 1%	Atmospheric: 100 °C, ≥18 h High-pressure: 120 °C, 0.15 MPa, 4 h	20–25	Temp.: 18–22 °C (forest environment) RH: 85–90% Key Practice: Utilizes forest canopy (density 0.6–0.8); natural climate control.	“Prevention first”. Physical methods primary (e.g., sticky boards, nets); no chemical pesticides during fruiting.
Henan Provincial Department of Agriculture and Rural Affairs, 2016	Henan (Temperate)	Facility-based (Various shed types)	1. Sawdust-based: Sawdust 79%, Wheat bran 18%, Corn meal 1.2%, Soybean meal 0.8%, Gypsum 1% 2. Corn Cob-based: Sawdust 49%, Corn cobs 30%, Wheat bran 20%, Gypsum 1%	High-pressure: 0.14–0.15 MPa, 1.5–2 h Atmospheric: 100 °C, 12–15 h + steaming > 8 h	23–25 (early) 21–23 (late)	Temp.: 16–20 °C RH: 85–95% Light: 200–400 Lux CO_2_: <0.1% Key Practice: Seasonal scheduling (spring/autumn).	Integrated methods. Pesticide use prohibited during fruiting; complies with GB/T 8321.
Sichuan Provincial Market Supervision Administration, 2019	Sichuan (Southwest Transitional)	Seasonal Bag Cultivation	1. Balanced Mix: Sawdust 30%, Cottonseed hulls 48%, Wheat bran 15% 2. Sawdust Dominant: Sawdust 78%, Wheat bran 20%, Gypsum 1%	Atmospheric: 100 °C, 12–15 h High-pressure: 121 °C, 1.5–2 h	22–25 (max ≤ 30)	Temp.: 16–20 °C RH: 90–95% Light: Primordia 50–150 Lux, Differentiation 200–400 Lux Key Practice: Utilizes agricultural by-products.	Prevention-focused. Chemical control complies with GB/T 8321; prohibited during fruiting.
Hebei Provincial Department of Agriculture and Rural Affairs, 2018	Hebei (Temperate)	Seasonal Bag Cultivation	1. Cottonseed Hull Dominant: Cottonseed hulls 90%, Wheat bran 9% 2. Cotton-Corn Mix: Cottonseed hulls 59%, Corn cobs 30%, Wheat bran 10%	Atmospheric: 100 °C, >12 h High-pressure: 0.14–0.15 MPa, 2–2.5 h	20–25	Temp.: 15–20 °C RH: 80–90% Light: 200–500 Lux CO_2_: ~0.03% (≤0.1%) Key Practice: Adapted to temperate fluctuations; uses local crop residues.	“Prevention first, comprehensive control”. Chemical as last resort.
Tibet Autonomous Region Market Supervision Administration, 2024	Tibet (Plateau)	Protected Agriculture (Greenhouse)	1. High Cottonseed Hull: Cottonseed hulls 88%, Wheat bran 10%, Quicklime 1%, Gypsum 1% 2. Wood-Cotton Mix: Broadleaf wood dust 44%, Cottonseed hulls 44%, Wheat bran 10%, Gypsum 1%	High-pressure: 0.12–0.14 MPa, 121–126 °C, 2–3 h	20–25	Temp.: 15–20 °C RH: 80–90% Key Practice: Protected cultivation (greenhouses) to buffer extreme plateau conditions; wall-style stacking.	“Prevention first, comprehensive control”. Agricultural & physical methods prioritized; chemical pesticides prohibited during fruiting.

Note: This table synthesizes key agronomic parameters extracted from official Chinese local technical reports (cited in-text as Technical Reports), which document the climate-zone-specific cultivation systems analyzed in this review. Representative substrate formulas are presented for each region to illustrate the diversity of local resource utilization. The parameters reflect long-term (≥5 years) agronomic practices recorded in these standards and provide practice-based reference for the cultivation systems described. This table is presented in accordance with the critical mapping and evidence synthesis requirements for scoping reviews [20]. Of the 15 local technical reports identified, eight representative standards were selected for inclusion.

## Data Availability

This study is a scoping review. The “data” analyzed consist of the published literature and technical standards cited within the article. All supporting sources are fully referenced and listed in the References section. No new primary datasets were generated.
